# Case Report: Perspective of a Caregiver on Functional Outcomes Following Bilateral Lateral Pectoral Nerve Cryoneurotomy to Treat Spasticity in a Pediatric Patient With Cerebral Palsy

**DOI:** 10.3389/fresc.2021.719054

**Published:** 2021-09-06

**Authors:** Jack Scobie, Paul Winston

**Affiliations:** ^1^Island Medical Program, University of British Columbia, Victoria, BC, Canada; ^2^Division of Physical Medicine and Rehabilitation, University of British Columbia, Victoria, BC, Canada; ^3^Canadian Advances in Neuro-Orthopedics for Spasticity Congress, Victoria, BC, Canada

**Keywords:** cryoneurotomy, spasticity, cerebral palsy, caregiver perspective, pediatrics

## Abstract

Spasticity is common and difficult to manage complication of cerebral palsy that significantly affects the function and quality of life of patients. This case study reports a 15-year-old male with quadriplegic cerebral palsy, Gross Motor Function Classification System 5 (GMFCS 5), who presented with significant bilateral adducted and internally rotated shoulders as a component of generalized spasticity. Spasticity in the lower limb of the patient had been treated with botulinum toxin A (BoNT-A) injections; however, the shoulder region was spared due to concerns of toxin spread and aspiration risk. Following diagnostic nerve blocks, the patient underwent bilateral cryoneurotomies of the right and left lateral pectoral nerves (LPNs) lasting 3.5 min for each lesion. One month after the cryoneurotomies, the range of motion (ROM) had improved from 86° to 133° on the right and 90° to 139° on the left. Improvements in ROM were retained at 9 months post-procedure. At 8.5 months following the cryoneurotomies, the caregiver reported improvements in upper body dressing, upper body washing, transferring, and the ability of the patient to remain sitting in his wheelchair for extended periods. Cryoneurotomy may be an effective procedure for improving shoulder ROM and specific functional outcomes for caregivers of patients with spasticity arising from cerebral palsy.

## Introduction

Spasticity is a common and important complication of cerebral palsy that has a significant impact on the quality of life and functional capacity of patients (1). Targeted management of muscle spasticity is a key aspect of patient care. Various therapeutic options are available for managing spasticity, typically consisting of a combination of pharmacological treatments and surgical or injectable modalities (2). The use of a mini-invasive percutaneous cryoneurotomy to induce disruption of the axon and myelin is an emerging technique for managing spasticity (3). There is substantial evidence of the efficacy of cryoanalgesia in the pain literature; however, literature outlining the use of cryoneurotomy for spasticity treatment is limited (3, 4). There is no literature available on functional outcomes of cryoneurotomy in the pediatric population (3). This sentinel case demonstrates quantitative improvements in range of motion (ROM) and qualitative improvements that have been reported from the caregiver of a patient who underwent bilateral pectoral cryoneurotomy to manage spasticity arising from cerebral palsy.

## Case Report

This study conforms to all Case Reports (CARE) guidelines and reports the required information accordingly (refer to Supplementary Material 2). The parent/caregiver provided informed consent for the publication of this study. A 14-year-old male with quadriplegic cerebral palsy, Gross Motor Function Classification System 5 (GMFCS), presented with problematic bilateral adducted and internally rotated shoulders as a component of generalized spasticity in the upper and lower limbs and cervical spasticity/dystonia. He had been treated with botulinum toxin A (BoNT-A) injections to various arm and leg muscles based on symptomatology. No BoNT-A had been injected into the shoulder girdle due to fears of toxin spread and aspiration risk. He had undergone surgical release of his hip adductors. The patient had repeated admissions to hospital intensive care for recurrent pneumonia and gastrointestinal bleeding with hematemesis, which affected his BoNT-A regimen. He had a generalized seizure disorder. He was referred to the multidisciplinary spasticity clinic for consideration of a novel cryoneurotomy procedure to counteract problematic tone in the upper extremities.

The physical examination revealed a greatly reduced shoulder ROM with abduction passively to 85° on the right and 90° on the left, with no active abduction. His Modified Ashworth Scores (MAS) were four, with a fixed end point. The elbows had minimal spasticity, and the wrists and fingers were held in a fist, but flexible, with contracture noted at the metacarpophalangeal joints. His parent/caregiver reported that the painful shoulder positions greatly affected his daily care needs, such as dressing, bathing, and sitting. Diagnostic anesthetic motor nerve blocks (DNBs) were performed to each of the right and left lateral pectoral nerves (LPNs). The DNB causes temporary nerve conduction cessation to differentiate between the presence of shoulder girdle muscle contracture necessitating surgical release due to musculotendinous retraction (an unsuccessful block) vs. a reducible deformity due to spastic muscle overactivity (a successful block). Under ultrasound guidance, the neurovascular bundles of the LPN were identified using a longitudinal orientation along the chest, four fingerbreadths below the coracoid process. Lidocaine (1.5 ml of 2%) was injected juxtaposed to each of the right and left LPNs at the undersurface of the pectoral major muscle (PMM). After the DNB, there was an improvement in passive ROM in shoulder abduction to 120° bilaterally and a reduction in spasticity on the MAS (refer to Supplementary Material 1). There was an observed reduction in facial grimacing and easing of heavy respirations with passive abduction.

The decision was then made to proceed to percutaneous cryoneurotomies of both the LPNs. The procedure was delayed until the medical stability of the patient improved. He was then 15-year old. The procedures were performed 10 days apart in an outpatient interventional suite. An aseptic technique was used with 2% chlorhexidine and betadine. The ultrasound-guided cryoneurotomy was performed using a Lloyd SL 2000 Neurostat (San Diego, CA, USA) with a 1.2-mm cryoprobe at −60°C placed through a #16 angio guide. E-stimulation was performed to confirm nerve contact at 0.8 mV. The ice ball was repositioned to contact the LPN at two spots along the nerve. Each lesion was treated for 3.5 min. Hemostasis was achieved using skin glue and a plaster bandage. There were no surgical complications with the procedure or complications reported by the caregiver following the procedure.

### Quantitative Results

**Figure 1:**
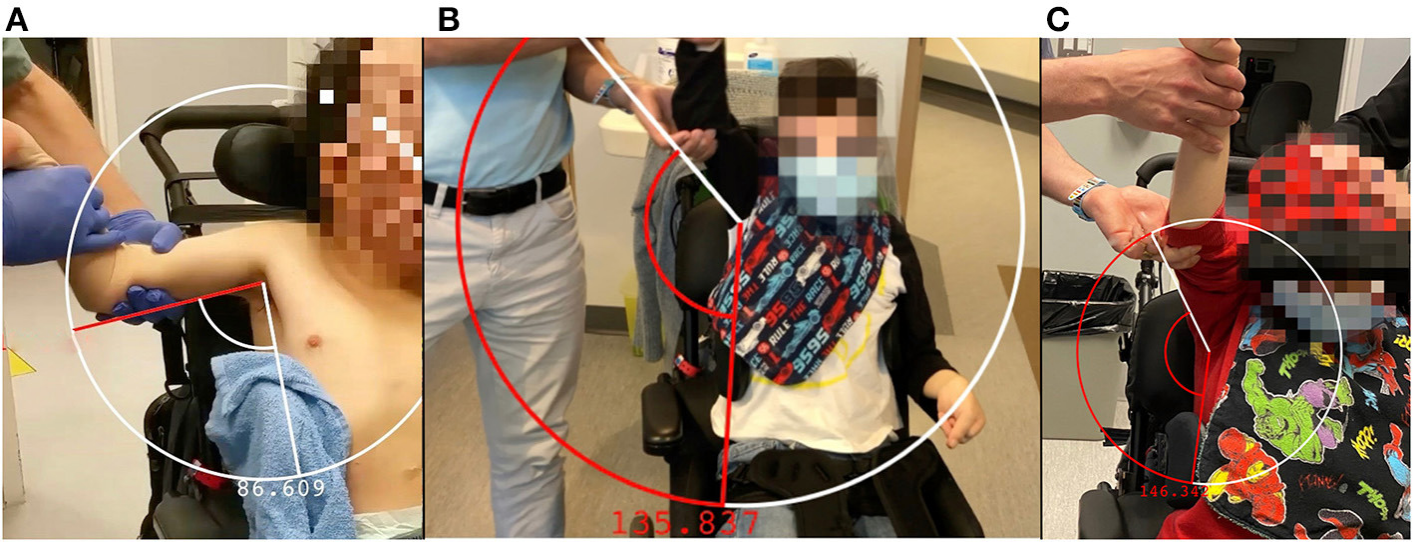
Abduction and arm position of the right arm prior to cryoneurotomy **(A)**, at 1 month following cryoneurotomy **(B)** and 9 months following cryoneurotomy **(C)**.

One month after the bilateral cryoneurotomies, the ROM in abduction had improved from 86 to 133° on the right and 90 to 139° on the left (Figure 1 and Supplementary Material 1). The MAS was reduced to two. He was next seen for follow-up at 9 months. The improvement in ROM noted a gain on the right to 146° and a reduction to 125° left (Figure 1 and Supplementary Material 1). The reduction in tone was to MAS 1+ within the available ROM.

### Qualitative Results

For this assessment, the Care and Comfort Caregiver Questionnaire (CareQ) was used. The CareQ is an assessment tool that has been adapted from the Caregiver Questionnaire (CQ), a questionnaire created in 1990 to assess children with spastic quadriplegic cerebral palsy prior to and following selective posterior rhizotomy (5, 6). In creating the CareQ, the CQ was modified to emphasize caregiver experience and goal setting for the child (5). The CareQ focuses on three functional areas, namely, personal care, position/transfers, and comfort (5). Functional outcomes are compared in these three areas prior to and following the procedure in a retrospective manner (Table 1). The caregiver of the patient was administered the CareQ over the phone following the procedure for 8.5 months. The patient is dependent on the caregiver to undertake all personal care tasks outlined in the questionnaire. At 8.5 months following the cryoneurotomies, there was an improvement in putting on shirts, taking off shirts, and washing the upper body of the patient. There was an improvement in the ability of the patient to remain sitting in a wheelchair for 3 h, the ease of transferring the patient into and out of the wheelchair, and the ease of applying orthotics. There were improvements in comfort levels during position changes while sitting in his wheelchair and while participating in school programs and physiotherapy.

**Table 1:** Results of CareQ were completed by the caregiver of a 15-year-old patient with cryoneurotomy who underwent bilateral pectoral cryoneurotomy.

**Personal care**	**Before cryoneurotomy**	**After cryoneurotomy**
Performing oral-facial hygiene	2	2
Putting on shirts	4	3
Taking off shirts	4	3
Putting on pants	3	3
Taking off pants	3	3
Changing incontinence pads or briefs	3	3
Cleaning buttocks or perineum with toileting	3	3
Washing upper body	4	2
Washing lower body	3	3
**Positioning/Transfers**		
How easy do you think it is for your child to remain sitting in a wheelchair for about 3 hours?	3	1
Ease of transferring your child into/out of wheelchair or other surfaces?	3	2
Ease of applying orthotics	4	3
**Comfort**		
How often do you think your child has had pain or discomfort during diaper or clothing changes?	N/A	N/A
How often do you think your child has had pain or discomfort during position changes?	3	1
How often do you think your child has had pain or discomfort while sitting in a wheelchair?	3	2
How often do you think pain or discomfort has prevented your child from participating in family activities?	0	0
How often do you think pain or discomfort has prevented your child from participating in school programs or community activities?	3	1
How often has your child had difficulty sleeping through the night?	3	3

*Scores range 1–5, with “1” defined as “very easy” and “5” defined as “impossible.” Abbreviation: CareQ, the Care and Comfort Caregiver Questionnaire*.

## Discussion

Spasticity arising from cerebral palsy is challenging to treat, and clinical approaches to management vary due to a lack of strong evidence to inform pharmacological therapy regimens (7). The field of spasticity management for patients with cerebral palsy may be amenable to novel therapies that have a clinical benefit. Traditional approaches to spasticity management consist of pharmacologic, surgical or neurolytic, and injectable options, such as botulinum toxin (2, 7–12). Pharmacological regimens may consist of diazepam, baclofen, or trihexyphenidyl (7–12). Cryoneurotomy percutaneously induces selective neurolysis of a motor nerve to manage spasticity, similar to other injectable and surgical modalities, such as partial neurotomy and chemodenervation by alcohol or phenol (2, 3). In cryoneurotomy, the axons and myelin of peripheral nerves are disrupted by the tip of the cryoprobe which may reach −70°; however, the epineurium is maintained allowing for nerve regeneration (3, 4, 13). Cryoneurotomy carries less risk of damage to surrounding tissue than phenol or alcohol chemodenervation (3). This procedure has been shown to have a clinically significant impact on spasticity reduction, even in cases refractory to other therapeutic strategies (3). Cryoneurotomy for the flexed elbow spasticity was shown to maintain the improved ROM and reduced MAS at a mean follow-up interval of 12.5 months in 11 patients including maintenance in the longest follow at over 2 years (14).

**Figure 2:**
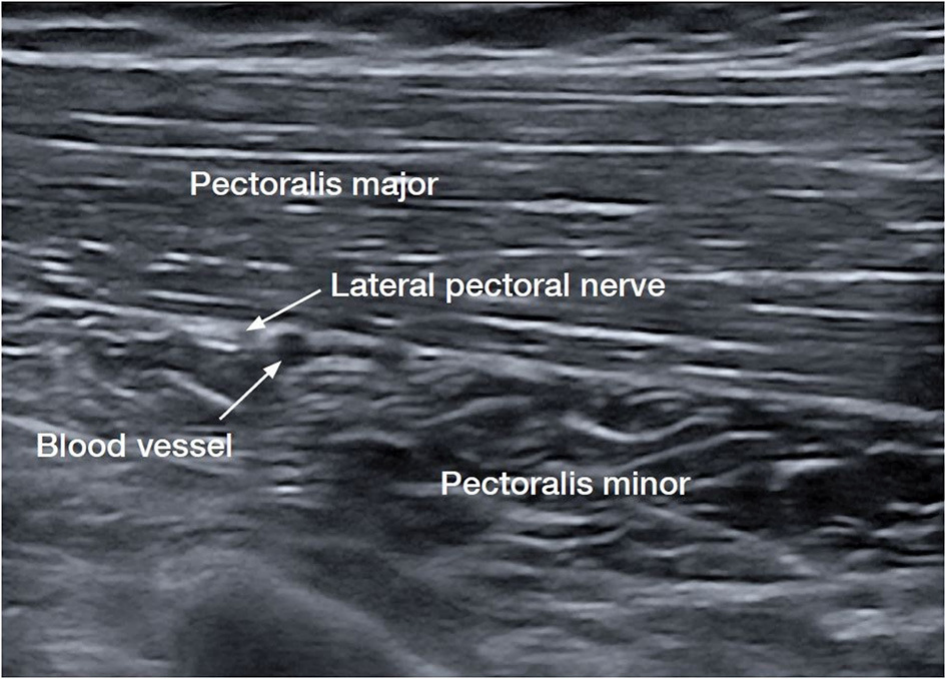
Ultrasound image of the lateral pectoral nerve (LPN), the dominant nerve of the pectoralis major muscle (PMM) (15, 16).

The LPN is the dominant innervation to the pectoralis major muscle (PMM). Anatomical studies have demonstrated that the LPN was found to have a highly consistent course after leaving the lateral trunk of the brachial plexus alongside the blood vessels on the undersurface of the pectoralis major in 100 consecutive patients (15, 16). The PMM is the largest muscle implicated in shoulder adduction and internal rotation (17). The LPN was shown to innervate both heads of the pectoralis major, (15) while the lower portion of the PMM has innervation from the medial pectoral and also from intercostal nerves (17). In contrast, the medial pectoral nerve is also thought to have a far less consistent course and is harder to consistently target (16, 17). It has been shown to dive below the pectoralis minor before rising along with the pectoralis major (18). The consistency of the LPN renders it the more easily identifiable nerve with ultrasound, a target for cryoneurotomy, and less deep and further away from the chest cavity (Figure 2) (18).

The passive abduction of the patient improved by an average of 55% within 6 weeks. At 9 months, the right nerve continued to show improvements in ROM. The left side reduced in ROM but maintained a 30° improvement compared to the measurement prior to the cryoneurotomy. The MAS remained reduced at 9 months with much greater ease in passive ROM of the shoulder. This is consistent with findings of cryoneurotomy for flexed elbow spasticity (14) and the tibial nerve (3).

In a non-verbal patient, discussion with the caregiver is necessary to identify goals and expectations. The frequent hospitalizations of this young patient for infection, respiratory compromise, seizures, and gastrointestinal bleeding made it challenging to attend the routine 3-month intervals for botulinum toxin, hence a longer-lasting procedure was the desired option. Due to the comorbidities of the patient, surgical interventions that carry a risk of toxin spread and respiratory compromise, such as BoNT-A injections, were avoided.

## Conclusion

This case demonstrates the impact that the emerging therapeutic procedure cryoneurotomy has on the LPN to reduce spasticity in a GMFCS 5 patient. Outcomes of cryoneurotomy were measured not only through improvement in spasticity and ROM but also through functional outcomes reported by the caregiver of the patient. Targeted cryoneurotomy to address spasticity in specific muscles may improve ROM and functionality in a variety of tasks, such as dressing, hygiene, transferring, and physiotherapy programs. Given the lack of standardized management in treating spasticity, there is a benefit to exploring novel procedures that may be efficacious in many patients, including those who are resistant to more traditional therapeutic options. Further research is necessary to determine how cryoneurotomy fits into the current practice of spasticity management.

## Data Availability Statement

The original contributions presented in the study are included in the article/Supplementary Material, further inquiries can be directed to the corresponding author/s.

## Ethics Statement

Written informed consent was obtained from the individual(s), and minor(s)' legal guardian/next of kin, for the publication of any potentially identifiable images or data included in this article.

## Author Contributions

PW provided the case study and patient care. JS provided the patient interview and outcome measures.

## Conflict of Interest

PW has funding for a clinical trial in adults with spasticity for cryoneurotomy, provided by Abbvie Allergan and Pacira. The remaining author declares that the research was conducted in the absence of any commercial or financial relationships that could be construed as a potential conflict of interest.

## Publisher's Note

All claims expressed in this article are solely those of the authors and do not necessarily represent those of their affiliated organizations, or those of the publisher, the editors and the reviewers. Any product that may be evaluated in this article, or claim that may be made by its manufacturer, is not guaranteed or endorsed by the publisher.

## Supplementary Material

Supplementary Material 1 (Multimedia) Demonstration of diagnostic nerve block on the lateral pectoral nerve (LPN) under ultrasound imaging followed by a demonstration of shoulder range of motion (ROM) prior to cryoneurotomy, at 1 month following cryoneurotomy, and 9 months following cryoneurotomy.

Supplementary Material 2 CARE checklist.
